# Missed Measles Immunisations Places Individuals and Communities at Risk: The Equity Argument for Including Measles in Under-Immunised Definitions

**DOI:** 10.3390/vaccines13020108

**Published:** 2025-01-22

**Authors:** Stefanie Vaccher, Moses Laman, Margie Danchin, Fiona Angrisano, Christopher Morgan

**Affiliations:** 1 Burnet Institute, 85 Commercial Rd, Melbourne, VIC 3004, Australia; 2 Papua New Guinea Institute of Medical Research, Boroko, National Capital District 111, Goroka P.O. Box 60, Papua New Guinea; 3 Vaccine Uptake Group, Murdoch Children’s Research Institute, Royal Children’s Hospital, 50 Flemington Road, Parkville, VIC 3052, Australia; 4 Department of Paediatrics, Faculty of Medicine, Dentistry and Health Sciences, University of Melbourne, 161 Barry St, Carlton, VIC 3010, Australia; 5 Department of General Medicine, Royal Children’s Hospital, 50 Flemington Road, Parkville, VIC 3052, Australia; 6 Jhpiego, The Johns Hopkins University Affiliate, 1615 Thames Street, Baltimore, MD 21231, USA; 7 Melbourne School of Population and Global Health, 207 Bouverie Street, Carlton, VIC 3053, Australia

**Keywords:** measles, vaccination, under-immunized, zero-dose, vaccine inequity

## Abstract

Background: Measles is consistently one of the leading causes of death from vaccine-preventable diseases in children, and cases and deaths have increased globally since 2019. While measles often serves as a ‘canary in the coalmine’ for health system weaknesses, global definitions of zero-dose and under-immunised children continue to centre on those who have missed diphtheria-tetanus-pertussis (DTP) containing vaccine. We propose that lack of receipt of measles vaccine is included in global definitions of ‘under-immunised’ children. Methods: We used publicly available WHO/UNICEF estimates of national immunization coverage (WUENIC) data to determine the number and proportion of children missing out on routine immunisations in each country globally in 2019 and 2022. We stratified countries by income status to further investigate inequalities in vaccine coverage between different countries. Results: In 2022, 50% more children missed out on their first dose measles-containing vaccine compared to DTP1, and 96% of these children resided in low-middle income countries (LMICs), highlighting the compounding inequities in measles immunisations globally. Furthermore, countries with the largest number of children missing out on DTP1 were not reflective of countries with the lowest measles immunisation coverage rates, suggesting targeted programs are needed to reach children who are missing out on measles vaccination. Recommendations: Given the high transmissibility and inequitable burden measles outbreaks pose to both at-risk individuals and communities, especially in LMICs, measles immunisation coverage should be included as a key metric when reporting and estimating the number of under-immunised children globally.

## 1. Introduction

Globally in 2019, there were an estimated 1.5 million deaths in children under five years of age attributed to vaccine-preventable diseases [1]. Measles is consistently one of the leading causes of death from vaccine-preventable diseases in children. It is highly transmissible and as such, requires 95% vaccination coverage across the population to reach herd immunity levels, compared to 90% for most other vaccines [2].

Although measles deaths declined by approximately 80% between 2000 and 2016, representing over 21 million deaths averted [1], in 2019 there were over 207,500 deaths from measles, an increase of almost 50% since 2016 [3].

Measles cases and deaths have continued to increase globally since 2019 [2]. This has been exacerbated by declines in routine immunisation coverage during the COVID-19 pandemic, especially in low- and middle-income countries (LMICs). As well as reductions in outreach services, clinic closures, and healthcare worker shortages [4], the politicisation of vaccination and growing vaccine misinformation have led to reduced trust in and uptake of routine childhood immunisations, which may have substantial ongoing future implications [5,6].

Only 83% of children globally received their first dose of measles-containing vaccine (MCV1) in 2022 [2]. Although this is an improvement from the low of 81% in 2021, prior to the COVID-19 pandemic, global MCV1 coverage had been stable around 85–86% since 2015. This represents an additional 2.7 million children globally who missed out on MCV1 in 2022 compared with 2019 [7].

The infectiousness of measles means it often serves as a canary in the coalmine for health system weaknesses, gaps in immunisation programs, and health inequities [8]. There is a dual risk for people who are not vaccinated against measles—both the lack of individual protection, but also the immunity gaps created in under-vaccinated communities that can lead to disruptive outbreaks.

Recent modelling from the World Health Organization (WHO) has shown that of the 154 million lives saved by immunisation since 1974, measles vaccination has accounted for over 60% of these [9]. Additionally, measles vaccination was shown to be the most substantial contributor to reductions in preventable mortality and morbidity.

The importance of achieving high coverage with two doses of measles vaccine has been recognised in the Sustainable Development Goals [10]. The Immunisation Agenda 2030 notes that “high coverage with measles vaccine is an indicator of a strong immunization programme, which may signal a solid foundation for primary health care services” [11]. It is also a key prevention indicator in the WHO Immunisation Joint External Evaluation (JEE) tool, which aims to understand the degree to which countries measure measles vaccination coverage to inform supplementary immunisation activities [12].

Gavi, the Vaccine Alliance, defines “zero-dose” as children who have not received their first dose of DTP (DTP1). Similarly, children are considered “under-immunised” if they have not received their third dose of diphtheria-tetanus-pertussis containing vaccine (DTP3) [13]. While these definitions are important in highlighting priority groups or settings that need to be targeted to ensure vaccine equity, further work is needed to understand if children who have not received DTP1 are representative of all children missing out on routine immunisations, including measles vaccination.

This analysis aims to compare the number of zero-dose and under-immunised children globally, based on the Gavi definitions, with the number and proportion of children who have missed out on measles immunisations in each country worldwide to estimate the additional burden of missed measles vaccination. In addition, equity issues with current immunisation program gaps will be highlighted to argue for the inclusion of measles immunisation coverage as part of the global definitions of ‘under-immunised’.

## 2. Methods

We used the publicly available 2022 WHO/UNICEF estimates of national immunization coverage (WUENIC) data to assess immunisation coverage for DTP1, DTP3, and MCV1 in each country globally in 2019 and 2022 [7]. These data were combined with the under-1 and under-2-year populations in each country in 2019 and 2022, which were extracted from the United Nations Population Estimates for children under five years [14].

Publicly available data were downloaded as comma-separated files and imported into Stata (v18, StataCorp, College Station, TX, USA) for analysis.

Country income status (low, lower-middle, upper-middle, and high income) was determined using the 2022 World Bank country classifications by income level [15].

The number of children missing each of DTP1, DTP3, and MCV1 in each country in 2019 and 2022 was calculated by multiplying the inverse of immunisation coverage with the number of children in the 12–23-month cohort in each country. This age cohort was chosen as there is no clear consensus on the age at which children should be considered ‘zero-dose’, but to ensure sufficient time had passed in which they could have been vaccinated, and to account for the differing ages at which children receive MCV1 globally [16].

Dropout is the difference in the proportion of children receiving one vaccine who do not go on to receive a second, later vaccine. For this analysis, dropout was calculated by country for three different indicators in 2022: the percentage of children receiving DTP1 minus the percentage of children receiving MCV1, the percentage of children receiving MCV1 minus the percentage of children receiving MCV2, and the percentage of children receiving DTP1 minus the percentage of children receiving DTP3. Change in coverage was calculated as 2022 coverage minus 2019 coverage for DTP1, DTP3, and MCV1.

Countries were also ranked on the number and proportion of children between 12 and 23 months who had not received DTP1 and MCV1. Ranks were calculated for each country based on DTP1 and MCV1 coverage, in ascending order, and number of children not vaccinated for DTP1 and MCV1, in descending order. If two countries shared a rank position for coverage, the higher number was assigned to both countries, and marked with an equal (“=”) sign. For example, if three countries (rank 14, 15, and 16) all reported 56% coverage for MCV1, they all received the rank of =16.

Wilcoxon signed-rank tests were used to compare the number of children who missed immunisations between different groups (e.g., by World Bank income strata).

## 3. Results

**Figure 1:**
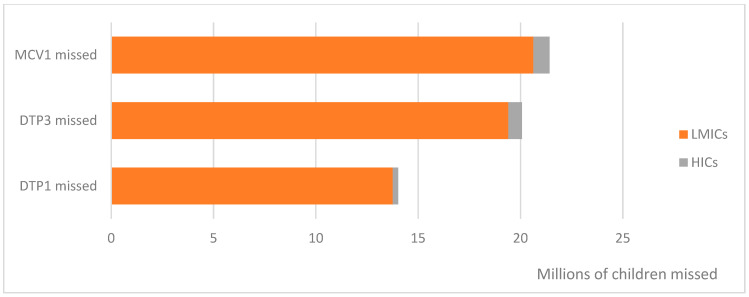
Number of children globally missing out on diphtheria-tetanus-pertussis (DTP) and measles-containing (MCV) vaccines in 2022, by World Bank strata. LMICs, low- and middle-income countries; HICs, high-income countries.

Globally in 2022, 14 million children missed out on their first dose of DTP vaccine, and 21 million children missed out on their first dose of MCV (Figure 1). This equates to 50% more children globally not receiving MCV1, compared to those missing out on DTP1. Almost all (96%) of these children resided in LMICs, highlighting the disproportionate burden resource-limited settings face in both preventing and responding to infectious disease threats.

**Figure 2:**
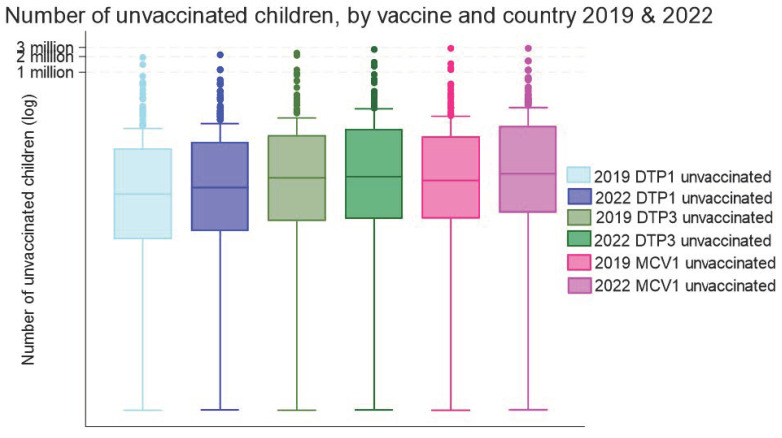
Median (IQR) number of children aged 12–23 months missing immunisations by country (N = 195) and vaccine, 2019 and 2022. DTP, diphtheria-tetanus-pertussis vaccine; MCV, measles-containing vaccine. Note: Data are log-transformed to aid visual interpretation.

The number of children missing MCV1 in 2022 was significantly higher than the number of children missing DTP1 or DTP3 (*p* < 0.001 for both, Wilcoxon signed-rank test). The number of children missing out on each of these three vaccines has also increased since 2019 (Figure 2).

Countries with the largest number of children aged 12–23 months who had missed immunisations are relatively consistent across DTP1, DTP3, and MCV1. In both 2019 and 2022, these were (in alphabetical order): Afghanistan, Angola, the Democratic Republic of Congo, Ethiopia, India, Indonesia, Nigeria, Pakistan, the Philippines, and Somalia. This is perhaps unsurprising given these countries are some of the most populous globally. The median coverage for DTP1 and MCV1 in these 10 countries was 76% and 64%, respectively.

Median MCV1 coverage globally (90%) was significantly lower than DTP1 (95%) or DTP3 (91%) coverage in 2022 (Table 1), and below the 95% coverage threshold required for herd immunity. When high-income countries were excluded from the analysis, median MCV1 coverage declined to 83%. Coverage of MCV1 also reduced more during the COVID-19 pandemic than coverage of DTP1 (*p* < 0.001, Wilcoxon signed-rank test).

Dropout is another way of measuring knowledge of vaccine schedules, accessibility, and service continuity and acceptability. Generally, immunisation coverage declines with subsequent doses as children age [17,18]. This is a particular concern for measles vaccination, where the second dose is not given until a child is 15–18 months of age or older.

Median DTP1 to MCV1 dropout in 2022 was 2 percentage points (interquartile range: 1–5%) in high-income countries (HICs), but 6 percentage points in LMICs (Table 2). There was further sustained dropout between MCV1 and MCV2 vaccination receipt in 2022. This highlights the additional challenge of continuing to engage with and reach children who have had some contact with the health system and previously received at least one vaccine in resource-limited settings. Further work is needed to identify and address barriers specific to this cohort.

Global efforts often focus on the number of zero-dose children [13,19]. This risks leaving behind smaller countries with higher proportions of un-immunised children, and increases the likelihood of future vaccine-preventable disease outbreaks.

When examining the top 20 countries ranked by the number of children who have not received DTP1 or MCV1, and by the percentage of children who have not received DTP1 or MCV1 (Table 3) some interesting differences emerge between countries. For instance, the United States appears to have the 19th-highest population of children who have not received MCV1 globally but is not featured in any of the other top 20 rankings.

Countries with the highest proportion of children missing DTP1 and MCV1 are not always aligned with countries with the highest number of children missing these vaccinations. This is especially the case in fragile or small island developing states, or countries with more rural and remote populations.

For instance, in 2022, 55% of children in Papua New Guinea missed out on DTP1 vaccination and 56% missed out on MCV1 [7]. Papua New Guinea is the largest country in the Pacific region by an order of magnitude, yet the number of children estimated to have missed out on DTP1 and MCV1 in 2022 was 134,812 and 137,263, respectively.

This compares to nearby Philippines, another lower-middle income country, which is generally recognised as having a high number of un-vaccinated children. The birth cohort is almost 10-fold larger than Papua New Guinea, so despite MCV1 coverage rates being 34% higher, the number of children who have not been vaccinated against measles is over five times larger, at almost 752,000.

**Figure 3:**
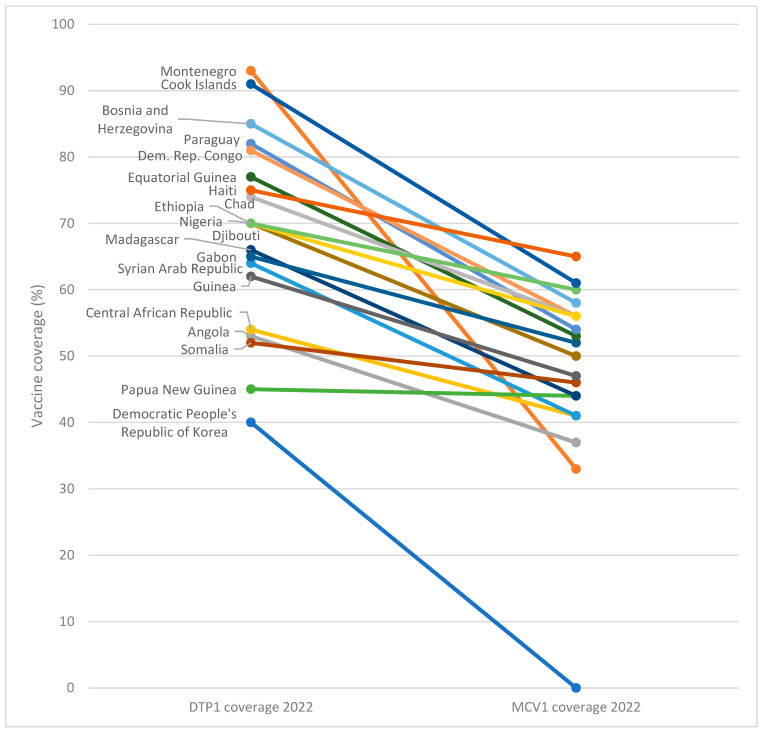
Difference in diphtheria-tetanus-pertussis (DTP) and measles-containing vaccine (MCV) coverage in 20 countries with lowest MCV1 coverage, 2022.

Using DTP1 coverage as a proxy for MCV1 coverage underestimates countries most at risk of measles outbreaks as coverage across the two vaccines is not always closely aligned. Out of the top 50 countries globally with the lowest MCV1 coverage in 2022, 20% (10 countries) did not appear on the list of the top 50 countries with the lowest DTP1 coverage (Figure 3). That means that one in five countries with the lowest MCV1 coverage is globally missed when we focus zero-dose efforts only on children who have not received DTP1.

Most critically, Montenegro was reported to have the second-lowest MCV1 coverage globally in 2022, but was not in the top 50 lowest DTP1 countries. This emphasises the need to measure and address measles-specific drivers and barriers to immunisation coverage. Notably, among the top 10 countries with the lowest coverage for MCV1 in 2022, the maximum coverage was 50%, with an average coverage across the 10 countries of 38%. For DTP1, average coverage among these 10 countries was 57% and maximum coverage reached 67%.

Of the 10 countries shown in Table 3 with the largest number of missed children, only two of these, Angola and Madagascar, appear in the list of the top 10 countries with the highest proportion of children who have not received MCV1. Although this analysis uses population-level data, and thus direct individual comparisons between children are not possible, examining trends at the country level can highlight areas of structural and systemic inequities within country immunisation programs.

There are many disparities in immunisation coverage within and between populations, and assessing just one vaccine cannot adequately represent the challenges with immunisation programmes. Thus, incorporating measles vaccine coverage into global definitions of under-immunised children can help to elucidate which populations are most at risk of future measles outbreaks and of being left behind in global efforts to increase vaccine coverage when the focus remains on receipt of DTP-containing vaccine.

## 4. Discussion

Globally, almost all children who have missed out on measles immunisation reside in LMICs, highlighting the inequities in measles immunisation coverage. Dropout between DTP1 and MCV1 was also three times higher in LMICs than HICs and was exacerbated during the COVID-19 pandemic.

Given the huge healthcare, social, and economic burden posed by measles outbreaks, combined with high transmissibility of measles and lower MCV1 coverage compared to DTP3 globally, we propose that the absence of a first dose of measles-containing vaccine is considered in global definitions of under-immunised children. This is important as coverage across DTP and measles vaccines is not always closely aligned, as shown in this study.

In countries with large populations, even with relatively high vaccine coverage, there can be large pockets of un- and under-immunised individuals. Reducing the risk of outbreaks by reducing clusters of susceptible individuals is critical, even in less populous settings [20]. The risk of an outbreak increases with a higher relative proportion of people who are unimmunised, especially for highly infectious diseases like measles.

Measles elimination remains a priority for the WHO and is a key indicator in the JEE, with all regions committing to eliminating measles but none yet achieving sustained elimination [2]. Given the differences identified in this analysis about where children live who have not been vaccinated with MCV1 compared to DTP1, routine data collection on the number and proportion of children at a national and sub-national level who have not been vaccinated with MCV1 is critical as a standalone indicator of immunisation program performance, progress towards elimination, and to assess the potential risk of future measles outbreaks.

In terms of equity, the enduring impacts of measles infection and outbreaks do not affect all individuals and populations equally. For instance, Papua New Guinea has reported a high incidence of subacute sclerosing panencephalitis (SSPE) following measles outbreaks, with increases in SSPE cases tracking closely with prior measles outbreaks [21]. In an under-resourced healthcare system, the double burden of vaccine-preventable disease outbreaks leading to extended morbidity and mortality is even more pronounced. This was also supported by recent WHO modelling highlighting that of the 9.0 billion life-years and 10.2 billion years of full health gained globally through vaccination since 1974, 5.7 and 5.8 billion years, respectively, are directly attributable to measles vaccination, more than all other vaccines combined [9].

Although receipt of DTP1 is important as it represents early contact with the healthcare system, previous research has shown that among children who have only received one vaccine, polio was the most likely vaccine to have been administered in low and lower-middle-income countries, and BCG in upper-middle income countries [22]. MCV was the least likely vaccine to have been received, and there were substantial disparities in DTP1 to MCV1 dropout by wealth quintile, highlighting further equity issues with MCV receipt.

Gavi uses DTP3 coverage as a proxy for overall health system performance and the ability to reach children with immunisations [13]. However, as this is administered to children around 3–4 months of age, it represents less sustained engagement with the healthcare system than measles vaccination, which is usually in the second year of life [23]. Thus, measles vaccination serves as a good proxy for ‘full immunisation’, ensuring that a child has received all critical age-appropriate vaccines and has ongoing access to healthcare.

Incorporating missed measles immunisations into global definitions and calculations of missed immunisations can also provide insights into the reasons behind low immunisation coverage. Utazi and colleagues [24] have identified that in areas with high DTP1 coverage but low DTP3 or MCV coverage, challenges are likely to be systematic. This includes inadequate follow-up, missed opportunities for immunisation, poor previous experience with health facilities, and low knowledge of immunisation schedules. Conversely, when both DTP1 and MCV coverage are low, structural challenges such as conflict, vaccine unavailability, and difficult terrain are likely contributing to poor immunisation rates.

This nuance exemplifies why consideration of measles vaccination coverage alongside currently used DTP indicators is useful to assess overall health system status, including in high-income settings. The inclusion of the United States on the list of countries with the highest number of un-vaccinated children for measles (Table 3) highlights that, while it has the third largest population of any country globally, there are issues specific to measles vaccination, or broader constraints around ongoing access and utilization of the health system that warrant further attention.

Attention to vaccine-specific barriers in resource-limited settings is also needed, as acceptance and willingness for uptake can differ between vaccines. This is especially the case for MCV, where previous research has identified fear of autism as an enduring piece of misinformation which can lead to hesitancy, despite being comprehensively rebuked [25]. Tools such as the WHO Behavioral and Social Drivers of Vaccination [26] or UNICEF’s Journey to Immunisation [27], which aim to understand the complex interplay of both access and acceptance barriers, can also be adapted for this purpose.

Focusing zero-dose efforts on countries with the largest number of un-immunised children may underestimate the risks of outbreaks in countries that have smaller populations but higher proportions of un- or under-vaccinated children. Additional immunisation challenges may also be present in conflict and fragile settings, or countries with greater gender disparities [13,28]. With increasing global mobility, outbreak risks are further exacerbated in more fragile health systems.

This analysis is limited by the lack of sub-national and demographic data publicly available in the WUENIC dataset. Additionally, as individual-level vaccination data were not available, some nuance may be lost during analysis. Further exploration of individual-level factors, particularly among under-immunised children, is warranted to better understand why some children are only partially immunized. The availability of high-quality data to provide evidence for gaps in MCV coverage at the local level is necessary to inform decision-making and enable corrective action. Routine immunisation data are already collected through the WHO/UNICEF Joint Reporting Form, but further utilisation of this data, including using MCV1 coverage as a key indicator for health equity and immunisation coverage can lead to renewed advocacy efforts to remove inequities around measles vaccination, at the same time as reducing the risk of future measles outbreaks.

Strategies that can lead to more equitable measles vaccination coverage globally may include transition from 10-dose to 5-dose vaccine vials, to reduce healthcare worker reluctance to open new vials for a few vaccines, or in coming years, implementation of measles microarray patches, which may reduce the need for cold chain and enable trained community members to administer vaccines.

Going forward, global health organisations and policymakers should emphasise the importance of measuring, reporting on, and advocating for the inclusion of measles immunisation coverage as a key metric when estimating numbers of un- and under-immunised children. This will be essential to create awareness of the need to increase measles vaccine coverage globally and reduce the number of children who currently miss out on life-saving immunisations each year. Re-focusing efforts on children who have missed out on measles immunisation will provide an opportunity to reach those who may have missed out on other childhood vaccinations or routine health checks and ensure the most vulnerable members of our communities can receive essential healthcare services.

## Figures and Tables

**Table 1:** Median (interquartile range) immunisation coverage and change in coverage 2019–2022.

Outcome of Interest	All Countries	Low-Middle Income Countries Only ^a^
MCV1 coverage 2022	90% (76–96%)	83% (71–93%)
DTP1 coverage 2022	95% (87–98%)	92% (82–97%)
DTP3 coverage 2022	91% (78–97%)	85% (75–94%)
Change in MCV1 coverage between 2019 and 2022	−2% (−7–0%)	−3% (−9–0%)
Change in DTP1 coverage between 2019 and 2022	0% (−3–0%)	−0.5% (−5–0%)

^a^ Country income status according to the World Bank 2022 country classifications [15]. DTP, diphtheria-tetanus-pertussis vaccine; MCV, measles-containing vaccine.

**Table 2:** Median (interquartile range) immunisation coverage–dropout in percentage points.

Outcome of Interest	All Countries	Low-Middle Income Countries Only ^a^
MCV1–MCV2 dropout 2022	5% (1–14%)	8% (1–17%)
DTP1–MCV1 dropout 2022	4% (1–11%)	6% (2–12%)
DTP1–DTP3 dropout 2022	3% (1–7%)	5% (2–10%)

^a^ Country income status according to the World Bank 2022 country classifications [15]. DTP, diphtheria-tetanus-pertussis vaccine; MCV, measles-containing vaccine.

**Table 3:** Top 20 countries ranked by number and percentage of children who have not received DTP1 or MCV1, and by income status.

Rank	Number of Children Without DTP1	Percentage of Children Without DTP1	Number of Children Without MCV1	Percentage of Children Without MCV1
1	Nigeria	Democratic People’s Republic of Korea	Nigeria	Democratic People’s Republic of Korea
2	Ethiopia	Papua New Guinea	Ethiopia	Montenegro
3	India	Somalia	Democratic Republic of the Congo	Angola
4	Democratic Republic of the Congo	Angola	India	Syrian Arab Republic (=5)
5	Philippines	Central African Republic	Pakistan	Central African Republic (=5)
6	Angola	Guinea	Angola	Madagascar (=7)
7	Indonesia	Syrian Arab Republic	Philippines	Papua New Guinea (=7)
8	Brazil	Gabon	Indonesia	Somalia
9	Pakistan	Madagascar	Brazil	Guinea
10	Mozambique	Mozambique	Madagascar	Djibouti
11	Somalia	Nigeria (=13)	Afghanistan	Gabon
12	Afghanistan	Ethiopia (=13)	Niger	Equatorial Guinea
13	Madagascar	Djibouti (=13)	Somalia	Paraguay
14	Cameroon	Venezuela *	Democratic People’s Republic of Korea	Ethiopia (=16)
15	Democratic People’s Republic of Korea	Philippines (=18)	Cameroon	Democratic Republic of the Congo (=16)
16	United Republic of Tanzania	Chad (=18)	United Republic of Tanzania	Chad (=16)
17	Chad	Ecuador (=18)	Cote d’Ivoire	Bosnia and Herzegovina
18	Myanmar	Libya (=18)	Chad	Nigeria
19	Guinea	Cameroon	United States	Cook Islands
20	South Africa	Haiti	Sudan	Cameroon

Key: White = low income; light blue = lower middle income; dark grey = upper middle income; black = high income, based on World Bank 2022 country classifications [15]; * no recent income data. DTP, diphtheria-tetanus-pertussis vaccine; MCV, measles-containing vaccine.

## Data Availability

Immunization data are publicly available from the WHO/UNICEF estimates of the national immunization coverage portal (https://worldhealthorg.shinyapps.io/wuenic-trends/, accessed 21 November 2023) and population data are publicly available from United Nations Population Estimates (https://population.un.org/wpp/Download/Standard/Population/, accessed 29 November 2023).
